# Solitary pelvic kidney encountered during laparoscopic colectomy

**DOI:** 10.4103/0972-9941.18998

**Published:** 2005-09

**Authors:** Kazuhiro Sakamoto, Yutaka Kojima, Ryohei Takeda, Kiyoshi Terai, Mitsuhiro Matsuda

**Affiliations:** Department of Coloproctological Surgery, Juntendo University, School of Medicine, Tokyo, Japan

**Keywords:** laparoscopic colectomy, operative complications, renal ectopia, unilateral renal agenesis

## Abstract

We report a case of solitary pelvic kidney encountered during laparoscopic colectomy. A 55-year-old man was admitted to undergo laparoscopic colectomy for an early sigmoid colon cancer, which had been detected after a polypectomy. The kidneys were not clearly visualized in their normal position by ultrasonography. During the operation, anomalous vessels in the presacral space and a mass covered with fatty tissue were identified. We converted the operation to a mini-laparotomy, and on performing intraoperative ultrasonography a solitary pelvic kidney was detected. An anterior resection was performed without operative complications. Laparoscopic ultrasonography (LUS) and hand-assisted laparoscopic surgery (HALS) should be considered as feasible adjuvants, when difficult situations arise during laparoscopic colectomy. In case of uncertainty about anatomical orientation or identification, it is prudent to convert to open surgery thereby preventing intraoperative complications such as injury to anomalous vessels or the ureter.

Renal ectopia results when the mature kidney fails to reach its normal position and is found in locations such as the pelvic, iliac, abdominal and thoracic regions.[1] We report a patient with a solitary pelvic kidney which was encountered during laparoscopic colectomy.

## CASE REPORT

A 55-year-old man was admitted to undergo laparoscopic colectomy for an early sigmoid colon cancer. He had been under treatment for chronic renal dysfunction and hypertension at another hospital, and was on medication for hypertension resulting from renal sclerosis. His serum level of creatinine was slightly high (2.11 mg/dl). Colonoscopy revealed a semi-protruding polyp (2 cm in diameter) in the sigmoid colon for which a polypectomy was carried out. Histopathologically, the polyp revealed adenocarcinoma invading the submucosa with lymphatic invasion. Abdominal ultrasonography did not visualize either kidney clearly. It was assumed that the kidneys had undergone atrophic changes due to the chronic renal dysfunction he suffered and no other imaging was undertaken preoperatively.

**Figure 1:**
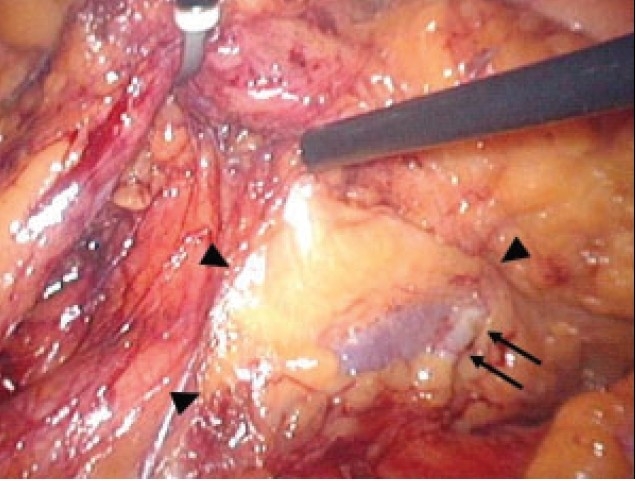
Laparoscopic findings: an anomalous vessel in the presacral space (arrows) and a round mass covered with fatty tissue (arrowheads) were identified

The operation was performed under pneumoperitoneum, and five ports were placed. Dissection of the mesentery was started at the bifurcation of the common iliac vessels. Although, the gonadal vessels were recognized in the correct posterior plane, the left ureter could not be identified on either side. The dissection was continued into the right side of the retroperitoneum. However, the plane between the fascia propria of the rectum and the presacral fascia was unclear and firmer than usual. Then anomalous vessels were identified in the presacral space. The vessels adhered to fatty tissue that was slightly different from the rectal mesenteric fatty tissue and formed a round mass [Figure 1]. We decided to convert the laparoscopy to an open procedure as an ectopic kidney was suspected. After a midline skin incision of about 8 cm was performed below the umbilicus, operative ultrasonography was carried out. It revealed a normal kidney pattern in the pelvis. Anterior resection was performed using the double stapling technique. Histopathologically, the examined lymph nodes were negative for metastasis.

**Figure 2:**
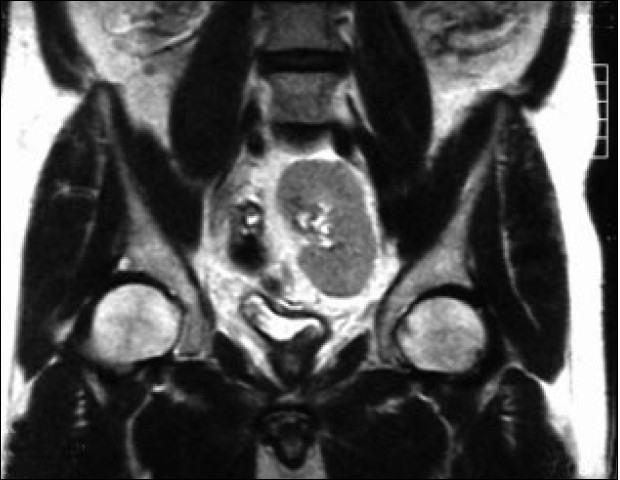
Postoperative MRI (Coronal *T*_2_-weighted image) showing a pelvic kidney with a normal nephrogram and the surrounding fatty tissue

No postoperative complications occurred and renal function tests showed no change. The postoperative magnetic resonance imaging (MRI) demonstrated a pelvic kidney with a normal nephrogram and surrounding fatty tissue [Figure 2].

## DISCUSSION

An ectopic kidney can be found in different locations, including the pelvic, iliac, abdominal and thoracic cavities.[1] A solitary pelvic kidney occurs in 1 of 22 000 autopsy cases.[2] They are usually discovered during an imaging study performed for other reasons. On the other hand, there was a report of an ectopic kidney misdiagnosed as an appendix mass or tumour leading to its removal.[3] In our case, the patient had previously not undergone any studies except renal function tests. Although, he underwent an ultrasonographic examination preoperatively, it was assumed that both the kidneys were atrophic as a consequence of his chronic renal dysfunction. To clarify the morphology of the kidneys and to obtain the anatomical information needed during laparoscopic surgery, other techniques, such as an abdominal CT without a contrast medium or an MRI should be used preoperatively.

It may be possible to encounter an asymptomatic ectopic kidney during surgery. What should be the appropriate approach when faced with such an eventuality? We decided to convert the laparoscopic colectomy to an open procedure for following reasons. First, the vascular pattern of the ectopic kidney was anomalous and this meant a likelihood of injuring the vessels that supplied the solitary pelvic kidney during the dissection between the pelvic kidney and the rectal mesentery. Gulsun et al[4] reported a right pelvic kidney fed by three arteries, which arose from bilateral common iliac arteries and from the ipsilateral internal iliac artery. The other reason was that we could not carry out laparoscopic ultrasonography (LUS) at that time. LUS is a simple and useful tool that compensates for the inability to physically palpate tissues.[5] Hand-assisted laparoscopic surgery (HALS), as well as LUS, should be considered as a feasible adjuvant when some tactile sensation is needed during laparoscopic surgery. HALS avoids intraoperative complications and reduces the need for conversion to open surgery due to the recovery of tactile feedback.

## CONCLUSIONS

LUS and HALS should be considered as feasible adjuvants when difficult situations arise during laparoscopic colectomy. However, we should not hesitate to convert laparoscopic colectomy to an open procedure, if deemed necessary.
